# Brain Structural Impairment in Spinocerebellar Ataxia Type 6: Not Restricted to the Cerebellum

**DOI:** 10.1007/s12311-026-02105-z

**Published:** 2026-10-29

**Authors:** Breno Kazuo Massuyama, Thiago Junqueira Ribeiro Rezende, Marcondes Cavalcante França, Orlando Graziani Povoas Barsottini, José Luiz Pedroso

**Affiliations:** 1 Department of Neurology and Neurosurgery, Federal University of Sao Paulo, Sao Paulo, Brazil; 2 Deparment of Neurology, State University of Campinas, 89-99 Vital Brasil Street, Campinas, Brazil

**Keywords:** Ataxia, Spinocerebellar ataxia, Diffusion tensor imaging, Magnetic resonance imaging

## Abstract

Spinocerebellar ataxias (SCA) refer to a group of autosomal dominant ataxic disorders that result from the degeneration of the cerebellum and its connections. SCA6 is described as the prototype of pure cerebellar ataxia, with preservation of other brain regions. However, the calcium receptor subunit affected in SCA6 appears to be ubiquitous in neurons throughout the brain. Additionally, there are observations of clinical involvement of non-cerebellar systems, structural cerebral damage and hypometabolism in various brain areas. To characterize the structural brain signature in SCA6 patients. Eighteen SCA6 patients underwent cross-sectional analyses using multimodal MRI-based techniques, which combined cerebral and cerebellar volumetric analyses with diffusion tensor imaging (DTI). Furthermore, we investigated whether structural abnormalities correlated with clinical findings. Individuals with SCA6, compared to non-ataxic controls, exhibited significant volumetric reduction in cerebellar white matter, cortex, and several lobules. There was increased axial diffusivity (AD) in the left inferior cerebellar peduncle, bilateral middle cerebellar peduncle, left superior cerebellar peduncle, bilateral midbrain, right cerebral peduncle, left superior corona radiata, bilateral fornix-stria terminalis, left sagittal stratum, left genu of the corpus callosum, right body of the corpus callosum, and left optic tract. Significant correlations were found between AD and the Inventory of Non-ataxia Symptoms Count, and between cerebellar volumetry and both the International Cooperative Ataxia Rating Scale and the Epworth Sleepiness Scale. We provided valuable insights into the possible extracerebellar structural abnormalities associated with SCA6. However, further studies, ideally including longitudinal follow-up, are needed before DTI-based analyses of cerebellar connections and supratentorial structures can be considered potential sources of biomarkers.

**Supplementary Information:**

The online version contains supplementary material available at https://doi.org/10.1007/s12311-026-02105-z.

## Introduction

Spinocerebellar ataxias (SCA) refer to a group of autosomal dominant ataxic disorders that result from the degeneration of the cerebellum and its afferent and efferent connections [1]. SCA6 (MIM: 183086) is the third most common subtype of SCA [2]. It is caused by a CAG expansion on chromosome 19, exon 47, in the gene CACNA1A, which codes for the α1A (Cav2.1) subunit. This subunit is the primary component of the voltage-dependent P/Q-type neuronal calcium channel, found in Purkinje cells (PC) [3]. In addition to SCA6, other disorders associated with the *CACNA1A* gene include familial hemiplegic migraine type 1 (MIM: 141500), episodic ataxia type 2 (MIM: 108500), and developmental and epileptic encephalopathy 42 (DEE42; MIM: 617106). Additional manifestations linked to *CACNA1A* encompass neuropsychiatric disorders, paroxysmal dystonia, epilepsy, and complex phenotypes involving various combinations of early developmental delay and epileptic encephalopathy [4]. SCA6 is described as the prototype of pure cerebellar ataxia, with preservation of other brain regions [1, 5]. It is characterized by cortical cerebellar degeneration involving PC [5]. However, the calcium receptor subunit affected in SCA6 appears to be ubiquitous in neurons throughout the brain [6].

Structural magnetic resonance imaging (MRI) of SCA6 patients reveals cerebellar atrophy, primarily axial, affecting the cerebellar vermis [7–10]. Cortical and cerebello-olivary atrophy can also be observed, but they are attributed to transsynaptic degeneration following the loss of PC [11]. Accordingly, SCA6 exhibits hypometabolism, in the anterior lobe of the cerebellar cortex and cerebellar vermis [12]. It is worth noting that, compared to healthy controls, SCA6 patients show hypometabolism not only in the cerebellar hemispheres but also in the brainstem, basal ganglia, and frontal, temporal, and occipital cerebral cortices [11]. In this sense, mild widespread cortical atrophy has been reported in SCA6 [13]. Additionally, SCA6 patients may present with mild external ophthalmoplegia, spasticity, or peripheral neuropathy [13]. Taken together, the observation of clinical involvement of non-cerebellar systems, structural cerebral damage and hypometabolism in these diverse brain areas suggests that SCA6 might not be a purely cerebellar syndrome.

To assess this hypothesis, we designed the current study to characterize the structural signature of the brain in a representative cohort of SCA6 patients through cross-sectional analyses using multimodal MRI-based techniques. These techniques combined cerebral and cerebellar volumetric analyses with diffusivity-based analyses. Additionally, we investigated whether the structural abnormalities correlated with the clinical findings.

## Materials and Methods

### Subjects’ Selection

Eighteen clinically and molecularly proven SCA6 patients who were evaluated at the Ataxia Unit, Federal University of Sao Paulo, agreed to participate in this study between 2020 and 2022. This study was approved by our Institutional Review Board under the number CAAE 36018820.8.0000.5505. All patients provided written informed consent. Patients with concomitant neurological disorders, those unable to undergo an MRI scan, or those who did not provide consent were excluded from the study. A control group of eighteen age-matched (± 2 years) and sex-matched healthy individuals underwent MRI scanning, and the results were compared with those of SCA6 patients. Individuals with any prior medical or family history of psychiatric or other neurological disorders were excluded.

### Clinical Protocol

For each patient, we gathered information on the age at onset, time from ataxia onset, the length of the (CAG) repeats in the longer allele, and the presence of any additional neurological symptoms. Disease severity was quantified using the “Scale for the Assessment and Rate of Ataxia” (SARA) and the “International Cooperative Ataxia Rating Scale” (ICARS) [14, 15]. For evaluation of non-motor and extracerebellar features, we applied “Inventory of Non-ataxia symptoms” (INAS), “REM Sleep Behavior Screening Questionnaire” (RBDSQ), “Epworth Sleepiness Scale” (ESS), and “Fatigue Severity Scale” (FSS) [16–19]. The INAS Count serves as a semiquantitative measure of extracerebellar involvement in SCA. RBDSQ is a numerical scale in which scores of five or higher suggest the presence of REM sleep behavior disorder (RBD). The ESS is an n-point scale where a threshold of ten or higher is used to diagnose excessive daytime sleepiness. FSS is an n-point scale where values greater than twenty-eight are indicators of the presence of fatigue. The clinical protocol was conducted on the same day as the imaging acquisition.

### MRI Acquisition

All subjects underwent high-resolution MRI acquisition on a 3T Phillips Achieva Scanner (Philips, Best, The Netherlands). The MRI scans were performed exclusively at the University of Campinas. Routine T2-weighted sequences were performed in all subjects to exclude unrelated abnormalities. A standard eight-channel head coil was used in all acquisition.

For gray matter (GM) analyses (FastSurfer and CerebNet), we used high-resolution T1 volumetric images of the brain with sagittal orientation, voxel matrix 240 × 240 × 180, voxel size 1 × 1 × 1mm^3^, repetition time (TR)/echo time (TE) 7/3.201 ms, and flip angle 8^o^.

For diffusion tensor imaging (DTI) multi-atlas analysis, we used a spin echo DTI sequence: 2 × 2 × 2 mm³ acquiring voxel size, interpolated to 1 × 1 × 2 mm3, reconstructed matrix 256 × 256, 70 slices, TE/TR 61/8,500 ms, flip angle 90^o^, 32 gradient directions, no averages, max b-factor = 1,000 s/mm^2^, and 6-minute scan.

For the routine T2-weighted sequence, we used a high-resolution T2 volumetric image of the brain with sagittal orientation, voxel matrix 176 × 176 × 321, voxel size 0.6 × 0.6 × 1.2 mm³, repetition time (TR)/echo time (TE) 7000/267 ms, and flip angle 90^o^.

### MRI Analysis

GM volumetry was performed using FastSurfer and CerebeNet softwares [20, 21], which provide optimal anatomical segmentations for cortical and subcortical segmentations, whereas white matter (WM) microstructure was assessed using a DTI multi-atlas approach [22], which has demonstrated high test–retest reproducibility and allows automated subject-level extraction of diffusion metrics [23]. Importantly, the anatomical regions evaluated in the T1-weighted volumetric analyses and those assessed using DTI are not directly equivalent, as the latter reflects WM tract integrity rather than GM anatomy.

#### FastSurfer

GM structures were evaluated using the FastSurfer software v.2.0.6, a deep learning-based tool for cerebral cortex and deep GM analysis [21]. FastSurfer shows improved performance when compared to the FreeSurfer pipeline v. 7.4.1 [24]. To accomplish that, the brain is parcellated using a convolutional neural network with U-Net architecture, followed by the standard FreeSurfer pipeline [20, 21]. In this step, two surfaces are created, the pial and white surfaces, using Gaussian filter with 10-mm full-width half-maximum to smooth both surfaces and enabling the fitting of a triangular mesh over them [25]. Cortical thickness is calculated as the shortest distance between the pial and white surface at each vertex across the cortical layer [20].

To better assess brainstem regions, we employed the FreeSurfer brainstem substructure segmentation pipeline, which allows us the identification of brainstem subunites such as, medulla oblongata, pons, midbrain, and superior cerebellar peduncle [26]. Such metrics were estimated using the cross-sectional subregion segmentation module within FreeSurfer (v.7.4.1) [14] that relied on a probabilistic Bayesian atlas of the brainstem [26].

Finally, estimated total intracranial volume (eTIV) was calculated using the standard FreeSurfer pipeline. This measure is derived from the affine transformation required to register each participant’s brain to the Talairach atlas and provides an automated estimate of global head size that is commonly used as a covariate in neuroimaging studies [27].

#### CerebNet

For cerebellar volumetry, we used the CerebNet software [24], v1.0, a secondary tool under the FastSurfer framework [21, 28]. CerebNet is also a deep learning-based solution dedicated to cerebellar segmentation. For better performance and generalizability, Cerebnet was trained using both controls and ataxic individuals [28].

#### White Matter Analysis

To assess WM microstructural changes, we employed the DTI multiatlas feature from “MRICloud” (MRICloud.org), a public web-based service for multicontrast imaging segmentation and quantification. Raw DTI-weighted images were first corrected for eddy currents and co-registered to remove subject motion using a 12-parameter affine transform [29, 30]. To quantify the DTI-parameters, we used a multivariate linear fitting. After that, the skull-stripping was performed by intensity threshold using the b = 0 image, a tool of RoiEditor software [31]. Next, we used the multicontrast LDDMM algorithm to register the atlas to the images and then the parcellation, which uses a DLFA algorithm [22, 32]. For whole-brain WM parcellation, it was used the Adult_168labels_8atlases_V1 library implemented in MRICloud, which comprises eight healthy atlas subjects aged 20–50 years and generates 168 anatomical structures. All analyses were performed in native space. Computations were processed on the Gordon cluster of XSEDE [33].

### Statistical Analysis

Before statistical analyses, data distribution was assessed using the Kolmogorov–Smirnov test. Most variables showed approximately normal distributions. Therefore, parametric tests were used for the analyses. Patients were compared to age- and sex-matched controls for all MRI-based analyses and demographical (age and sex) analyses. To assess group differences, we used the ANCOVA test and the Pearson correlation coefficient to evaluate the correlations between MRI-based parameters and clinical metrics. Age, sex, and eTIV were treated as confounding effects and, hence, their effects were removed from all variables. All statistical tests were corrected for multiple comparisons using the Bonferroni method, with statistical significance defined as a Bonferroni-corrected *p*-value < 0.05. The Bonferroni correction was applied separately to each imaging metric according to the number of regions analysed, including 62 cortical thickness measures, 21 subcortical volumes, 30 cerebellar volumes, 5 brainstem subsegmentation volumes, and 110 regions for each diffusion metric (FA, AD, MD, and RD). To distinguish between corrected and uncorrected significance levels, *p*-values are reported as *p*-Bonferroni when adjusted using the Bonferroni correction and as *p* when no correction was applied. Additionally, we calculated effect sizes (ES) for all statistically significant results using Cohen’s d formula. According to established conventions, we classified ES values as follows: 0.2 as small, 0.5 as moderate, 0.8 as large, and > 1.2 as very large [34]. For all imaging techniques, statistical analyses were based on regions of interest (ROI), and we evaluated all anatomical labels provided for each tool. Statistical analysis was done using the Matlab R2017b software (https://www.mathworks.com/products/matlab.html).

## Results

### Demographics and Clinical Characteristics

The demographic, genetic, and clinical data of the study population are summarized in Table 1. In short, this is a cohort of 18 SCA6 patients with late disease onset (mean = 53.3 years; SD = 9.1), long disease duration (mean = 13.0 years; SD = 7.3), and short CAG repeat length (mean = 22.1; SD = 1.2). Of note, the CAG repeat length was unavailable for six patients, and one patient had both expanded alleles (20 and 24), with only the allele with the larger expansion being considered for the statistics.

**Table 1:** Demographic, clinical and genetic data of the ataxic study participants

	SAC6 patients (*N* = 18)	Control group (*N* = 18)
Age (years); mean ± SD	67.7 ± 9.2	67.4 ± 9.0
Sex, n (male/female)	6/12	6/12
Disease duration; mean ± SD	13.0 ± 7.3	N/A
Age at the onset of symptoms	53.3 ± 9.1	N/A
CAG repeat length, long allele*✝	22.1 ± 1.2	N/A
SARA; mean ± SD	13.3 ± 6.7	N/A
ICARS; mean ± SD	37.0 ± 17.1	N/A
INAS Count; mean ± SD	1.7 ± 1.0	N/A
RBDSQ; mean ± SD	4.0 ± 26.6	N/A
ESS; mean ± SD	5.9 ± 4.0	N/A
FSS; mean ± SD	34.0 ± 13.0	N/A

*ESS* Epworth Sleepiness Scale, *FSS* Fatigue Severity Scale, *ICARS* International Cooperative Ataxia Rating Scale, *INAS* Inventory of Non-ataxia symptoms, *RBDSQ* REM Sleep Behavior Screening Questionnaire, *SARA* Scale for the Assessment and Rating of Ataxia, *SD* standard deviation

*CAG repeat length unavailable for six patients. ✝One patient had both expanded alleles (20 and 24): only the allele with the higher repeat number was considered for the statistics

Regarding ataxia severity, the SARA range was 2.5 to 25 (mean = 13.30) and the ICARS range was 8 to 66 (mean = 37). Moreover, we observed a high frequency of non-motor features in SCA6 patients beyond cerebellar signs: INAS Count (mean = 1.77), RBDSQ (mean = 4), ESS (mean = 5.94), and FSS (mean = 34).

### MRI Analysis

Comprehensive statistics, including coefficients and adjusted p-values for the MRI metrics, are provided in the Supplementary Appendix (Table S1). Table 2 summarizes the statistically significant findings after the Bonferroni correction, along with corresponding effect sizes for the MRI metrics.

**Table 2:** Summary of statistically significant findings

MRI analysis	Structures	Control (mean±SD)	SCA6 (mean±SD)	p-Bonferroni	ES
Brainstem (mm^3^)	Superior Cerebellar Peduncle Volume	242.118 ± 40.303	172.621 ± 30.663	0.000104542	1.94
Cerebellar volumetry (mm^3^)	Left Cerebellar White Matter	12623.066 ± 2074.875	8880.170 ± 1679.946	1.25238E-05	1.98
Cerebellar volumetry (mm^3^)	Left Cerebellar Cortex	41384.771 ± 5416.682	25032.531 ± 7168.378	1.08535E-07	2.57
Cerebellar volumetry (mm^3^)	Right Cerebellar White Matter	12537.681 ± 2111.617	8749.668 ± 1628.447	5.60043E-06	2.01
Cerebellar volumetry (mm^3^)	Right Cerebellar Cortex	41152.228 ± 4990.159	24694.078 ± 6801.507	4.0212E-08	2.76
Cerebellar volumetry (mm^3^)	Left Cerebellar Lobules I–IV	2975.023 ± 386.591	1577.387 ± 366.191	3.57407E-10	3.71
Cerebellar volumetry (mm^3^)	Right Cerebellar Lobules I–IV	2799.739 ± 409.131	1593.507 ± 291.927	1.13053E-08	3.39
Cerebellar volumetry (mm^3^)	Left Cerebellar Lobule V	3039.151 ± 533.196	1749.564 ± 385.121	2.05059E-07	2.77
Cerebellar volumetry (mm^3^)	Right Cerebellar Lobule V	3087.045 ± 480.428	1702.837 ± 445.542	7.28042E-09	2.99
Cerebellar volumetry (mm^3^)	Left Cerebellar Lobule VI	6054.641 ± 1322.557	3515.219 ± 998.774	2.70534E-05	2.17
Cerebellar volumetry (mm^3^)	Vermis Lobule VI	1349.434 ± 193.153	737.288 ± 250.404	1.65227E-07	2.74
Cerebellar volumetry (mm^3^)	Right Cerebellar Lobule VI	6113.156 ± 1222.695	3354.003 ± 1000.848	4.81225E-06	2.47
Cerebellar volumetry (mm^3^)	Left Crus I	9358.412 ± 1515.687	5628.533 ± 1730.109	5.98383E-06	2.29
Cerebellar volumetry (mm^3^)	Right Crus I	9583.282 ± 1379.348	5503.793 ± 1573.842	2.26966E-07	2.76
Cerebellar volumetry (mm^3^)	Left Crus II	5451.586 ± 1194.713	3439.820 ± 1410.457	0.007007573	1.54
Cerebellar volumetry (mm^3^)	Left Lobule VIIB	4078.530 ± 558.725	2233.921 ± 937.392	1.81474E-05	2.39
Cerebellar volumetry (mm^3^)	Right Lobule VIIB	3861.637 ± 529.194	2084.764 ± 812.284	6.15756E-06	2.59
Cerebellar volumetry (mm^3^)	Left Lobule VIIIA	4469.154 ± 971.583	2800.545 ± 1123.643	0.001606936	1.59
Cerebellar volumetry (mm^3^)	Right Lobule VIIIA	4301.945 ± 795.147	2575.680 ± 941.587	0.000128987	1.98
Cerebellar volumetry (mm^3^)	Left Lobule VIIIB	3190.283 ± 453.121	2037.248 ± 418.417	1.77411E-07	2.64
Cerebellar volumetry (mm^3^)	Right Lobule VIIIB	3177.730 ± 505.791	1946.092 ± 482.429	1.58775E-07	2.49
Cerebellar volumetry (mm^3^)	Left Flocculonodular lobe	462.939 ± 52.953	229.748 ± 93.644	5.9754E-07	3.06
Cerebellar volumetry (mm^3^)	Right Flocculonodular lobe	438.084 ± 43.420	224.452 ± 110.859	3.82776E-05	2.54
Cerebellar volumetry (mm^3^)	Vermis Lobule VII	643.584 ± 134.638	320.737 ± 151.625	8.04835E-05	2.25
Cerebellar volumetry (mm^3^)	Vermis Lobule VIII	1635.151 ± 247.146	1074.830 ± 364.256	0.000595502	1.80
Cerebellar volumetry (mm^3^)	Total Vermis	4788.525 ± 606.333	3050.001 ± 872.279	4.11503E-06	2.31
Axial diffusivity (x 10^-3^ mm^2^/s)	Left Inferior Cerebellar Peduncle	1.325 ± 0.110	1.472 ± 0.065	0.008503619	1.62
Axial diffusivity(x 10^-3^ mm^2^/s)	Left Superior Cerebellar Peduncle	1.699 ± 0.101	1.852 ± 0.102	0.01919825	1.51
Axial diffusivity (x 10^-3^ mm^2^/s)	Left Superior Corona Radiata	1.059 ± 0.072	1.148 ± 0.044	0.019781995	1.48
Axial diffusivity (x 10^-3^ mm^2^/s)	Left Fornix/Stria Terminalis	1.364 ± 0.118	1.545 ± 0.120	0.014262828	1.52
Axial diffusivity (x 10^-3^ mm^2^/s)	Left Sagittal Stratum	1.231 ± 0.077	1.337 ± 0.036	0.008612211	1.76
Axial diffusivity (x 10^-3^ mm^2^/s)	Left Middle Cerebellar Peduncle	1.165 ± 0.079	1.312 ± 0.061	0.000975981	2.09
Axial diffusivity (x 10^-3^ mm^2^/s)	Left Genu of the Corpus Callosum	1.531 ± 0.105	1.640 ± 0.064	0.047600773	1.26
Axial diffusivity (x 10^-3^ mm^2^/s)	Left Midbrain	1.210 ± 0.087	1.325 ± 0.078	0.007627304	1.40
Axial diffusivity (x 10^-3^ mm^2^/s)	Right Cerebral Peduncle	1.424 ± 0.048	1.519 ± 0.065	0.022314728	1.64
Axial diffusivity (x 10^-3^ mm^2^/s)	Right Fornix/Stria Terminalis	1.357 ± 0.071	1.469 ± 0.098	0.006555161	1.30
Axial diffusivity (x 10^-3^ mm^2^/s)	Right Middle Cerebellar Peduncle	1.158 ± 0.063	1.276 ± 0.050	0.001039935	2.08
Axial diffusivity (x 10^-3^ mm^2^/s)	Right Body of the Corpus Callosum	1.530 ± 0.076	1.636 ± 0.063	0.038608546	1.51
Axial diffusivity (x 10^-3^ mm^2^/s)	Right Midbrain	1.283 ± 0.086	1.394 ± 0.097	0.047932541	1.20
Axial diffusivity (x 10^-3^ mm^2^/s)	Left Optic Tract	1.794 ± 0.142	1.968 ± 0.187	0.021950161	1.05

Mean and standard deviations are provided for control and SCA6 groups

*ES * Cohen's d effect size, *p-Bonferroni * Bonferroni-corrected p-value

**Fig. 1:**
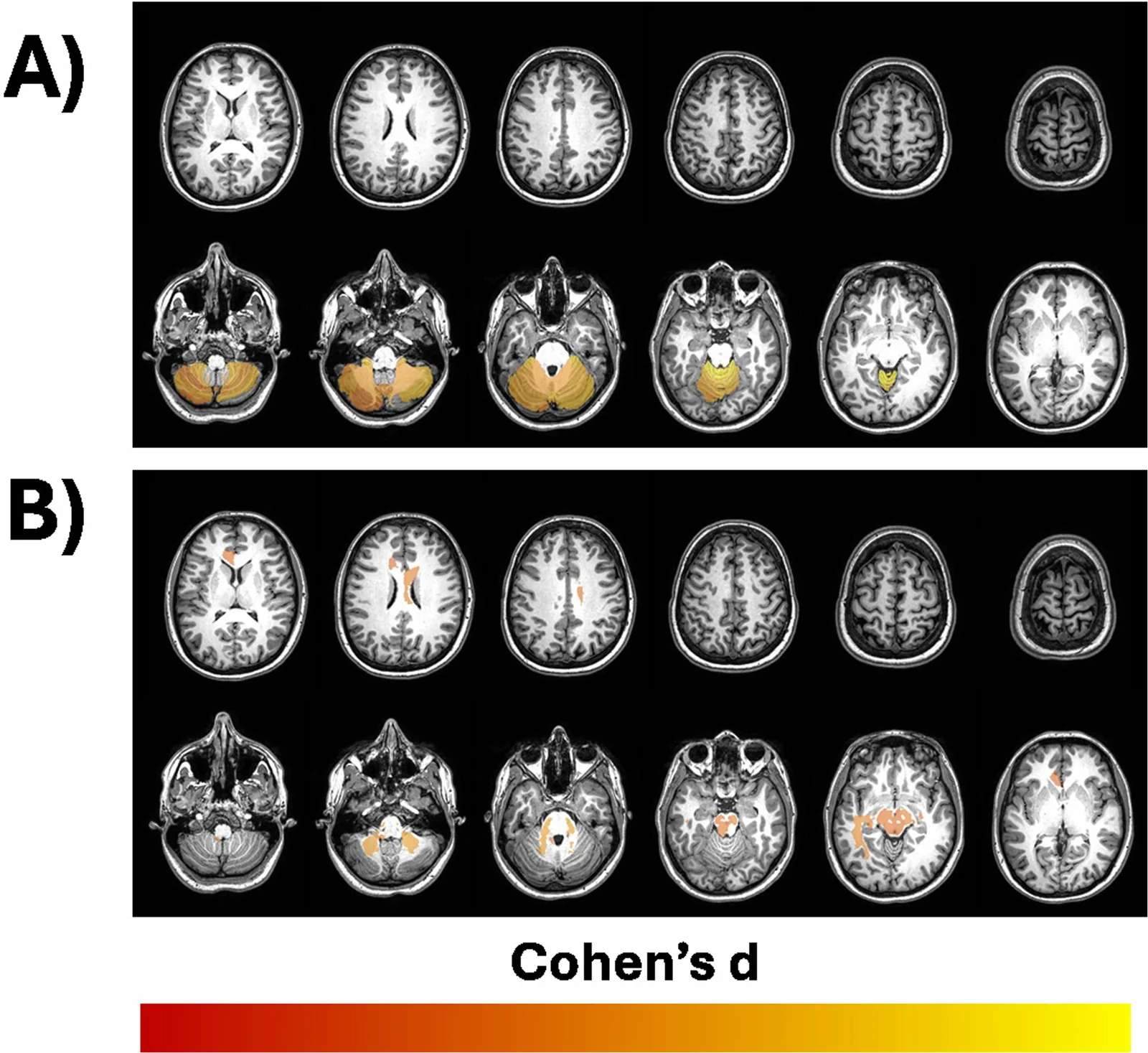
Cerebellar and cerebral structural damage in patients with SCA6, compared to age- and gender-matched controls, after Bonferroni adjustment for multiple comparisons. (**A**) Cerebellar GM and WM volumetry obtained using CerebNet. (**B**) Cerebral WM damage refers to the AD results from the DTI Multi-Atlas obtained using MRICloud. AD: axial diffusivity; DTI: diffusion tensor imaging; GM: gray matter; WM: white matter

After Bonferroni correction, the cerebellar analyses revealed significant volumetric reduction in bilateral WM (left and right: *p*-Bonferroni < 0.001), in whole cerebellar cortex of the left and right hemisphere (left and right: *p*-Bonferroni < 0.001) and in several cerebellar lobules: bilateral lobules I-IV (left and right: *p*-Bonferroni < 0.001), bilateral lobule V (left and right: *p*-Bonferroni < 0.001), bilateral lobule VI (left and right: *p*-Bonferroni < 0.001), vermis VI (*p*-Bonferroni < 0.001), bilateral crus I (left and right: *p*-Bonferroni < 0.001), left crus II (*p*-Bonferroni < 0.001), bilateral lobule VIIb (left and right: *p*-Bonferroni < 0.001), bilateral lobule VIIIa (left and right: *p*-Bonferroni < 0.001), bilateral lobule VIIIb (left and right: *p*-Bonferroni < 0.001), bilateral lobule X (left and right: *p*-Bonferroni < 0.001), vermis VII (*p*-Bonferroni < 0.001), and vermis VIII (*p*-Bonferroni < 0.001) (Fig. 1). All these structures exhibited very large ES (> 1.5).

After Bonferroni correction no significant group differences were observed in cortical thickness, brainstem volumes, or deep GM volumes. However, although these findings did not remain statistically significant after correction for multiple comparisons, large effect sizes were observed in the corpus callosum (ES > 1.1), the bilateral accumbens area (ES > 0.9), and the superior cerebellar peduncles (ES > 1.9), all showing trend-level effects with uncorrected p-values < 0.05.

### DTI Analysis

After Bonferroni correction, there were increased axial diffusivity (AD) in the left inferior cerebellar peduncle, bilateral middle cerebellar peduncle, left sagittal stratum, left genu of the corpus callosum, bilateral midbrain, right cerebral peduncle, right fornix-stria terminalis, right body of the corpus callosum, and left optic tract (*p*-Bonferroni < 0.001); left superior cerebellar peduncle, left superior corona radiata, and left fornix-stria terminalis (*p*-Bonferroni = 0.001) (Fig. 1). All these structures exhibited at least large ES (> 1.0).

Increased mean diffusivity (MD) values were found in left inferior cerebellar peduncle (*p* < 0.030), left superior cerebellar peduncle (*p* < 0.038), left superior corona radiata (*p* < 0.002), left fornix stria terminalis (*p* < 0.042), left sagittal stratum (*p* < 0.011), left middle cerebellar peduncle (*p* < 0.016), left midbrain (*p* < 0.001), right midbrain (*p* < 0.020), and left optic tract (*p* < 0.003).

After Bonferroni correction for multiple comparisons, no significant group differences were observed in mean diffusivity (MD), fractional anisotropy (FA), or radial diffusivity (RD).

### Correlations Between MRI Metrics and Clinical Scores

**Fig. 2:**
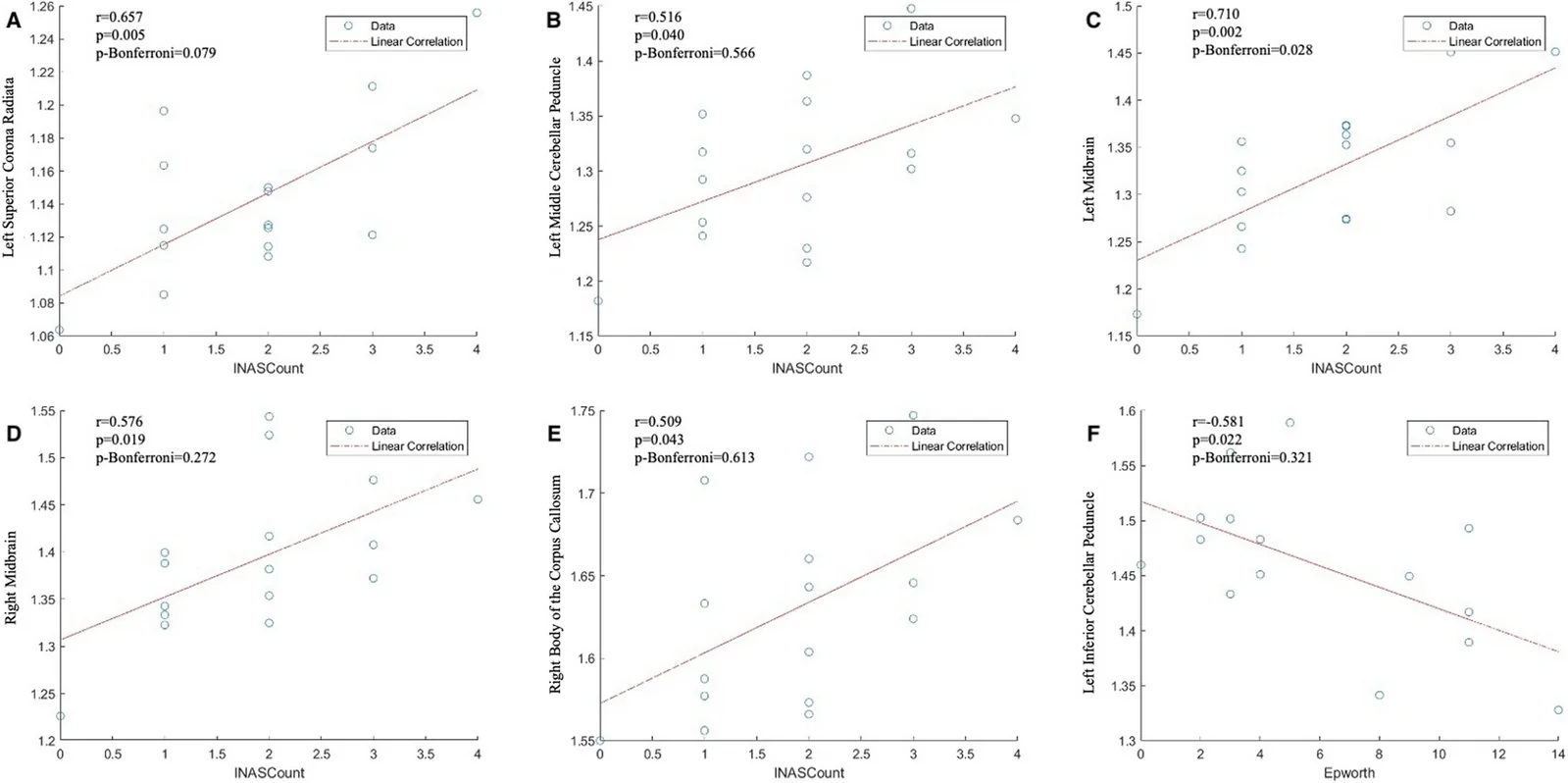
Correlations between AD and INAS Count in left superior corona radiata (**A**), left middle cerebellar peduncle (**B**), left midbrain (**C**), right midbrain (**D**), and right body of the corpus callosum (**E**). After Bonferroni correction, only the association with the left midbrain remained significant. Correlations between AD and ESS in left inferior cerebellar peduncle (**F**). AD: axial diffusivity; ESS: Epworth Sleepiness Scale; INAS: Inventory of Non-ataxia symptoms; p-Bonferroni = Bonferroni-corrected p-value

**Fig. 3:**
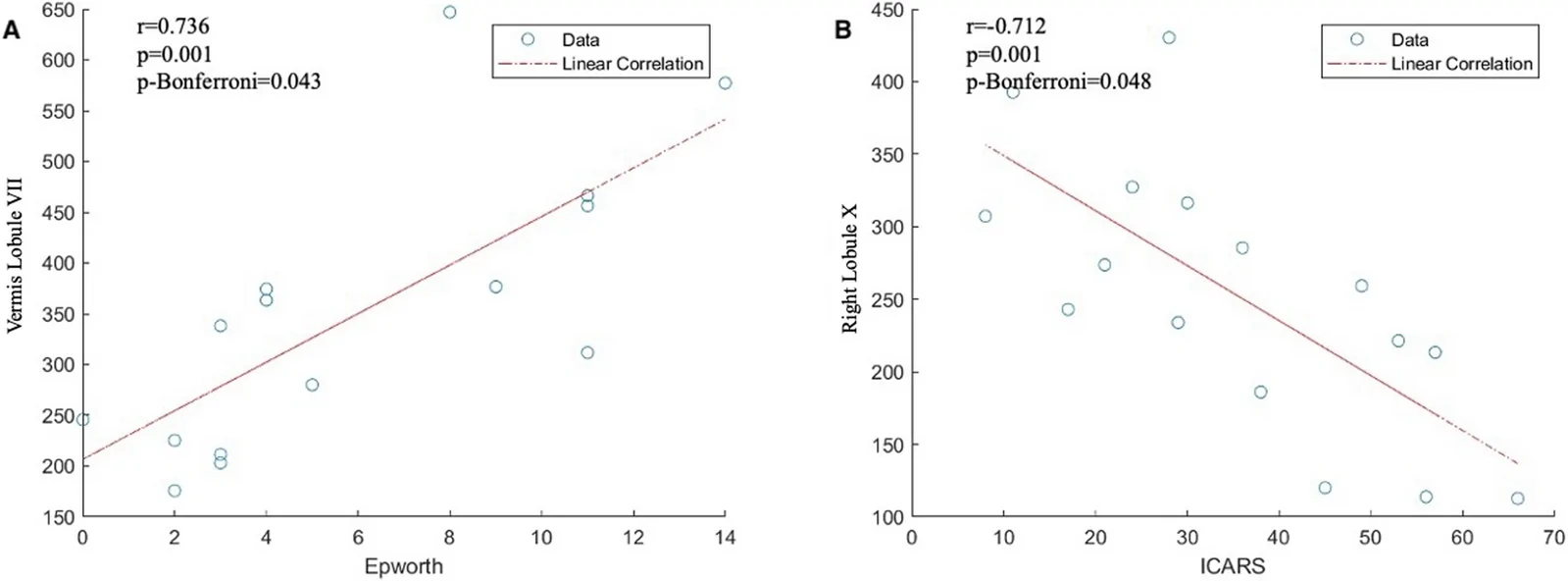
Correlations between cerebellar volumetric reduction and ESS in vermis lobule VII (**A**), and ICARS in right lobule X (**B**). ESS: Epworth Sleepiness Scale; ICARS: International Cooperative Ataxia Rating Scale; p-Bonferroni = Bonferroni-corrected p-value

After Bonferroni correction, significant correlations were observed between AD in the left midbrain and INAS count (*p*-Bonferroni = 0.028) (Fig. 2). In addition, cerebellar volumetry demonstrated significant Bonferroni-corrected correlations with ICARS in the right lobule X and with ESS in vermis lobule VII (Fig. 3). Although not significant after correction for multiple comparisons, but showing *p* < 0.05, additional associations were observed between AD in the left superior corona radiata, left middle cerebellar peduncle, right midbrain, and right body of the corpus callosum and INAS count, as well as between AD in the left inferior cerebellar peduncle and ESS.

## Discussion

In this study, we present the clinical and radiological findings from a cohort of eighteen patients with SCA6. Overall, we identified robust evidence that the pathological involvement in SCA6 is not confined solely to the cerebellum, with some of these alterations showing clinical correlations. Consistent with expectations, we observed marked volumetric changes across cerebellar regions, each exhibiting large effect sizes. Moreover, and in line with our hypothesis of extracerebellar involvement in SCA6, the DTI analysis revealed disruptions within cerebellar pathways as well as previously unrecognized extracerebellar alterations, most notably within structures involved in cerebellum–cortical circuits and across several supratentorial regions implicated in cognitive functions.

Cerebellar ataxias encompass a heterogenous group of conditions that include both sporadic and genetic etiologies [35]. Among the autosomal dominant cerebellar ataxias, the most common group is SCA, with over fifty-one distinct types clinically described (SCA1–51) [36]. In our Ataxia Unit in Brazil, among 1,332 patients with several types of ataxias, 326 are related to SCA, with SCA6 being the fifth most frequent (5.21%) [37]. SCA6 is referred to as pure cerebellar ataxia, with preservation of other brain regions [1, 5]. However, the calcium receptor subunit affected in SCA6 appears to be widespread in neurons throughout the brain [6]. Recent studies suggest that cerebellar degeneration becomes widespread, potentially leading to transneuronal degeneration in other brain regions [5, 38–41]. Moreover, PET-FDG demonstrated multiple-regional brain hypometabolism, including cerebellar hemispheres, brainstem, cortical regions, and basal ganglia [42]. Nevertheless, the presence of hypometabolism in different areas of the brain does not necessarily reflect neuronal degeneration. Instead, it could simply indicate metabolic dysfunction in neurons that are still structurally intact or subclinical neuropathological changes [42]. Falcon et al. demonstrated that, in SCA6, functional connectivity between regions of the cerebral cortex and cerebellum is accompanied by DTI metrics reflecting structural changes in the cerebral and cerebellar peduncles [43]. To illustrate the possible extracerebellar and non-motor changes in SCA6, within our cohort of SCA6 patients, one patient exhibited parkinsonism and dopaminergic dysfunction on a DAT (dopamine active transporter) scan [44]. Additionally, we reported that patients with SCA6 experience more frequent respiratory events and sleep apnea compared to a control group [45]. In this context, one might speculate that SCA6 would have alterations in the spinal cord. In fact, spinal cord abnormalities appear early and worsen progressively in SCA1, SCA2, and SCA3. However, SCA6 does not exhibit any morphometric abnormalities in the spinal cord [46, 47].

AD refers to diffusivity along a WM fiber tract, making it most relevant in regions where axons are coherently oriented without fiber crossings [48]. Increased isotropic diffusion appears to enhance both radial and axial diffusivity in chronic diseases marked by significant axonal damage [49]. Our patient group exhibited increased AD values compared with the control group, not only within cerebellar-connecting structures but also across several supratentorial regions. In addition, a significant correlation was observed between AD and the INAS Count in the midbrain. The INAS Count provides a straightforward quantitative index of extracerebellar neurological involvement, derived by summing the number of non‑ataxia signs present based on the absence or presence of 16 predefined features: areflexia, hyperreflexia, extensor plantar response, spasticity, paresis, amyotrophy, fasciculations, myoclonus, rigidity, chorea, dystonia, resting tremor, sensory symptoms, brainstem oculomotor abnormalities, urinary dysfunction, and cognitive impairment [9]. Nevertheless, although increased AD may be associated with neurodegeneration, this correlation might not reflect non-ataxia symptoms in SCA6 and may instead be attributable to the older age of patients with SCA6 [50].

Despite that, AD maps may not consistently align with the direction of the underlying fiber tract [48]. On the other hand, MD is a more reliable DTI metric for assessing multiple fiber populations or complex fiber geometries [51]. Additionally, MD shows a closer relationship to neurite density compared to FA, AD, or RD measures [52]. It is also a valuable tool for identifying and quantifying WM damage in neurodegenerative conditions, particularly in SCA [53]. Collectively, although the limited sample size and the fact that many findings did not survive correction for multiple comparisons, our results suggest the possibility of extracerebellar structural changes in SCA6.

Cerebellar activity plays a role in numerous aspects of sensorimotor control and motor learning [54]. Nevertheless, the involvement of the cerebellum in cognitive functions is still debated. There are cerebellar pathways that connect to the frontal cortex through the cerebello-ponto-thalamo-cortical connections. Consequently, the loss of cerebellar efferents to the frontal cortex could lead to cognitive changes [55]. Individuals with SCA6 exhibit notable impairments in attention and executive functions [56]. There is evidence that a network composed of the amygdala, various cortical regions, the hippocampus, basal forebrain, the nucleus accumbens, the stria terminalis, and the striatum participates in the modulation of memory consolidation [57]. Moreover, a previous study demonstrated a correlation between cognitive deficits and the MD of the stria terminalis in SCA2 [58]. Although not significant after correction for multiple comparisons, but showing uncorrected p-values < 0.05, we also observed increased MD values in the stria terminalis and the striatum. These findings suggest that a similar pathophysiological correlation between cognitive changes and these structures may also be present in SCA6. Conducting motor-independent cognitive tests in patients with purely cerebellar ataxias is crucial to elucidate the cerebellum’s role in cognition. Identifying non-motor and extracerebellar signs in SCA patients is key to the preclinical characterization of these diseases [42].

This study uncovers new aspects of SCA6, though the findings are preliminary and should be considered within the context of the following limitations. First, we did not establish a correlation between the radiological changes and the CACNA1A gene expression in the brain. Therefore, it remains unclear whether the extracerebellar findings result from local alterations directly induced by the mutated gene or if they indicate selective transneuronal degeneration. Second, considering that this is a cross-sectional study, it is not possible to determine if the non-motor and extra-cerebellar features developed prior to the ataxia. Further longitudinal studies are necessary to confirm these findings. Third, a further limitation of the study is the small sample size enrolled, which is also characterized by short CAG repeat lengths. Inclusion of individuals with longer repeats would likely provide more informative data. Fourth, the Bonferroni correction adjusts p-values to reduce the risk of Type I errors in multiple statistical tests. However, it has been criticized for potentially undermining sound statistical judgment and increasing the risk of Type II errors due to its conservative nature, especially as the number of comparisons increases.

## Conclusion

In conclusion, we provided valuable insights into the extracerebellar structural abnormalities associated with SCA6, fundamentally challenging the conventional understanding of SCA6 as a purely cerebellar ataxia. It remains unclear whether the findings, restricted to WM, indicate discrete extracerebellar features of SCA6 or rather reflect engagement of the complex cerebro-cerebellar networks involved in motor, cognitive, and affective processing. From a clinical standpoint, identifying biomarkers is essential for drug development aimed at treating or slowing the disease’s progression. Considering this, the MRI-derived metrics described here should be evaluated in longitudinal studies to determine their potential as imaging biomarkers in SCA6 and to further investigate the broader impact of SCA6 on cognitive and non-motor function.

## Supplementary Information

Below is the link to the electronic supplementary material.

Supplementary Material 1 (XLSX 54.7 KB)

## Data Availability

No datasets were generated or analysed during the current study.

## References

[1] Schöls L, Bauer P, Schmidt T, Schulte T, Riess O. Autosomal dominant cerebellar ataxias: clinical features, genetics, and pathogenesis. Lancet Neurol. 2004;3(5):291–304. 10.1016/S1474-4422(04)00737-9. 15099544

[2] Hersheson J, Haworth A, Houlden H. The inherited ataxias: genetic heterogeneity, mutation databases, and future directions in research and clinical diagnostics. Hum Mutat. 2012;33(9):1324–32. 10.1002/humu.22132. 22689585

[3] Zhuchenko O, Bailey J, Bonnen P, Ashizawa T, Stockton DW, Amos C, et al. Autosomal dominant cerebellar ataxia (SCA6) associated with small polyglutamine expansions in the alpha 1A-voltage-dependent calcium channel. Nat Genet. 1997;15(1):62–9. 10.1038/ng0197-62. 8988170

[4] Indelicato E, Boesch S. From Genotype to Phenotype: Expanding the Clinical Spectrum of CACNA1A Variants in the Era of Next Generation Sequencing. Front Neurol. 2021;12:639994. 10.3389/fneur.2021.639994. PMC7960780 33737904

[5] Ishikawa K, Watanabe M, Yoshizawa K, Fujita T, Iwamoto H, Yoshizawa T, et al. Clinical, neuropathological, and molecular study in two families with spinocerebellar ataxia type 6 (SCA6). J Neurol Neurosurg Psychiatry. 1999;67(1):86–9. 10.1136/jnnp.67.1.86. PMC1736420 10369828

[6] Hillman D, Chen S, Aung TT, Cherksey B, Sugimori M, Llinás RR. Localization of P-type calcium channels in the central nervous system. Proc Natl Acad Sci U S A. 1991;88(16):7076–80. 10.1073/pnas.88.16.7076. PMC52236 1651493

[7] Lukas C, Schöls L, Bellenberg B, Rüb U, Przuntek H, Schmid G, et al. Dissociation of grey and white matter reduction in spinocerebellar ataxia type 3 and 6: a voxel-based morphometry study. Neurosci Lett. 2006;408(3):230–5. 10.1016/j.neulet.2006.09.007. 17005321

[8] Butteriss D, Chinnery P, Birchall D. Radiological characterization of spinocerebellar ataxia type 6. Br J Radiol. 2005;78(932):694–6. 10.1259/bjr/73834093. 16046419

[9] Satoh JI, Tokumoto H, Yukitake M, Matsui M, Matsuyama Z, Kawakami H, et al. Spinocerebellar ataxia type 6: MRI of three Japanese patients. Neuroradiology. 1998;40(4):222–7. 10.1007/s002340050571. 9592791

[10] Murata Y, Kawakami H, Yamaguchi S, Nishimura M, Kohriyama T, Ishizaki F, et al. Characteristic magnetic resonance imaging findings in spinocerebellar ataxia 6. Arch Neurol. 1998;55(10):1348–52. 10.1001/archneur.55.10.1348. 9779664

[11] Koeppen AH. The pathogenesis of spinocerebellar ataxia. Cerebellum. 2005;4(1):62–73. 10.1080/14734220510007950. 15895563

[12] Oh M, Kim JS, Oh JS, Lee CS, Chung SJ. Different subregional metabolism patterns in patients with cerebellar ataxia by 18F-fluorodeoxyglucose positron emission tomography. PLoS ONE. 2017;12(3):e0173275. 10.1371/journal.pone.0173275. PMC5358749 28319124

[13] Schöls L, Krüger R, Amoiridis G, Przuntek H, Epplen JT, Riess O. Spinocerebellar ataxia type 6: genotype and phenotype in German kindreds. J Neurol Neurosurg Psychiatry. 1998;64(1):67–73. 10.1136/jnnp.64.1.67. PMC2169927 9436730

[14] Braga-Neto P, Godeiro-Junior C, Dutra LA, Pedroso JL, Barsottini OGP. Translation and validation into Brazilian version of the Scale of the Assessment and Rating of Ataxia (SARA). Arq Neuropsiquiatr. 2010;68(2):228–30. 10.1590/s0004-282x2010000200014. 20464290

[15] Maggi FA, Braga-Neto P, Chien HF, Gama MTD, Rezende Filho FM, Saraiva-Pereira ML, et al. Cross-cultural adaptation and validation of the International Cooperative Ataxia Rating Scale (ICARS) to Brazilian Portuguese. Arq Neuropsiquiatr. 2018;76(10):674–84. 10.1590/0004-282X20180098. 30427507

[16] Jacobi H, Rakowicz M, Rola R, Fancellu R, Mariotti C, Charles P, et al. Inventory of Non-Ataxia Signs (INAS): validation of a new clinical assessment instrument. Cerebellum. 2013;12(3):418–28. 10.1007/s12311-012-0421-3. 23090211

[17] Stiasny-Kolster K, Mayer G, Schäfer S, Möller JC, Heinzel-Gutenbrunner M, Oertel WH. The REM sleep behavior disorder screening questionnaire–a new diagnostic instrument. Mov Disord. 2007;22(16):2386–93. 10.1002/mds.21740. 17894337

[18] Johns MW. A new method for measuring daytime sleepiness: the Epworth sleepiness scale. Sleep. 1991;14(6):540–5. 10.1093/sleep/14.6.540. 1798888

[19] Toledo FO, Junior WM, Speciali JG, Sobreira CFDR. PND66 Cross-Cultural Adaptation and Validation of the Brazilian Version of the Fatigue Severity Scale (FSS). Value Health. 2011;14(7):A329–30. 10.1016/j.jval.2011.08.532.

[20] Fischl B, Dale AM. Measuring the thickness of the human cerebral cortex from magnetic resonance images. Proc Natl Acad Sci U S A. 2000;97(20):11050–5. 10.1073/pnas.200033797. PMC27146 10984517

[21] Henschel L, Conjeti S, Estrada S, Diers K, Fischl B, Reuter M. FastSurfer - A fast and accurate deep learning based neuroimaging pipeline. NeuroImage. 2020;219:117012. 10.1016/j.neuroimage.2020.117012. PMC7898243 32526386

[22] Tang X, Yoshida S, Hsu J, Huisman TAGM, Faria AV, Oishi K, et al. Multi-contrast multi-atlas parcellation of diffusion tensor imaging of the human brain. PLoS ONE. 2014;9(5):e96985. 10.1371/journal.pone.0096985. PMC4014574 24809486

[23] Rezende TJR, Campos BM, Hsu J, Li Y, Ceritoglu C, Kutten K, et al. Test-retest reproducibility of a multi-atlas automated segmentation tool on multimodality brain MRI. Brain Behav. 2019;9(10):e01363. 10.1002/brb3.1363. PMC6790328 31483562

[24] Henschel L, Kügler D, Reuter M, FastSurferVINN. Building resolution-independence into deep learning segmentation methods-A solution for HighRes brain MRI. NeuroImage. 2022;251:118933. 10.1016/j.neuroimage.2022.118933. PMC9801435 35122967

[25] Fischl B, Salat DH, Busa E, Albert M, Dieterich M, Haselgrove C, et al. Whole brain segmentation: automated labeling of neuroanatomical structures in the human brain. Neuron. 2002;33(3):341–55. 10.1016/s0896-6273(02)00569-x. 11832223

[26] Iglesias JE, Van Leemput K, Bhatt P, Casillas C, Dutt S, Schuff N, et al. Bayesian segmentation of brainstem structures in MRI. NeuroImage. 2015;113:184–95. 10.1016/j.neuroimage.2015.02.065. PMC4434226 25776214

[27] Buckner RL, Head D, Parker J, Fotenos AF, Marcus D, Morris JC, et al. A unified approach for morphometric and functional data analysis in young, old, and demented adults using automated atlas-based head size normalization: reliability and validation against manual measurement of total intracranial volume. NeuroImage. 2004;23(2):724–38. 10.1016/j.neuroimage.2004.06.018. 15488422

[28] Faber J, Kügler D, Bahrami E, Heinz LS, Timmann D, Ernst TM, et al. CerebNet: A fast and reliable deep-learning pipeline for detailed cerebellum sub-segmentation. NeuroImage. 2022;264:119703. 10.1016/j.neuroimage.2022.119703. PMC9771831 36349595

[29] Woods RP, Grafton ST, Holmes CJ, Cherry SR, Mazziotta JC. Automated image registration: I. General methods and intrasubject, intramodality validation. J Comput Assist Tomogr. 1998;22(1):139–52. 10.1097/00004728-199801000-00027. 9448779

[30] Zhuang J, Hrabe J, Kangarlu A, Xu D, Bansal R, Branch CA, et al. Correction of eddy-current distortions in diffusion tensor images using the known directions and strengths of diffusion gradients. J Magn Reson Imaging. 2006;24(5):1188–93. 10.1002/jmri.20727. PMC2364728 17024663

[31] Li X, Jiang H, Yue L, Mori S. Johns Hopkins University. http://www.MriStudio.org or http://www.kennedykrieger.org

[32] Jiang H, van Zijl PCM, Kim J, Pearlson GD, Mori S. DtiStudio: resource program for diffusion tensor computation and fiber bundle tracking. Comput Methods Programs Biomed. 2006;81(2):106–16. 10.1016/j.cmpb.2005.08.004 16413083

[33] Towns J, Cockerill T, Dahan M, Foster I, Gaither K, Grimshaw A, et al. XSEDE: Accelerating Scientific Discovery. Comput Sci Eng. 2014;16(5):62–74. 10.1109/MCSE.2014.80.

[34] Cohen J. Statistical power analysis for the behavioral sciences. 2nd ed. Hillsdale, N.J: L. Erlbaum Associates; 1988.

[35] Klockgether T. Sporadic ataxia with adult onset: classification and diagnostic criteria. Lancet Neurol. 2010;9(1):94–104. 10.1016/S1474-4422(09)70305-9. 20083040

[36] Tan D, Wei C, Chen Z, Huang Y, Deng J, Li J, et al. CAG Repeat Expansion in THAP11 Is Associated with a Novel Spinocerebellar Ataxia. Mov Disord. 2023;38(7):1282–93. 10.1002/mds.29412. 37148549

[37] Massuyama BK, Gama MTD, Silva TYT, Braga-Neto P, Pedroso JL, Barsottini OGP. Ataxias in Brazil: 17 years of experience in an ataxia center. Arq Neuropsiquiatr. 2024;82(8):1–8. 10.1055/s-0044-1787800. 38964341

[38] Seidel K, Siswanto S, Brunt ERP, den Dunnen W, Korf HW, Rüb U. Brain pathology of spinocerebellar ataxias. Acta Neuropathol. 2012;124(1):1–21. 10.1007/s00401-012-1000-x. 22684686

[39] Gierga K, Schelhaas HJ, Brunt ER, Seidel K, Scherzed W, Egensperger R, et al. Spinocerebellar ataxia type 6 (SCA6): neurodegeneration goes beyond the known brain predilection sites. Neuropathol Appl Neurobiol. 2009;35(5):515–27. 10.1111/j.1365-2990.2009.01015.x. 19207264

[40] Reetz K, Costa AS, Mirzazade S, Lehmann A, Juzek A, Rakowicz M, et al. Genotype-specific patterns of atrophy progression are more sensitive than clinical decline in SCA1, SCA3 and SCA6. Brain. 2013;136(Pt 3):905–17. 10.1093/brain/aws369. 23423669

[41] Rüb U, Brunt ER, Petrasch-Parwez E, Schöls L, Theegarten D, Auburger G, et al. Degeneration of ingestion-related brainstem nuclei in spinocerebellar ataxia type 2, 3, 6 and 7. Neuropathol Appl Neurobiol. 2006;32(6):635–49. 10.1111/j.1365-2990.2006. 17083478

[42] Soong B, Liu R, Wu L, Lu Y, Lee H. Metabolic characterization of spinocerebellar ataxia type 6. Arch Neurol. 2001;58(2):300–4. 10.1001/archneur.58.2.300 11176970

[43] Falcon MI, Gomez CM, Chen EE, Shereen A, Solodkin A. Early Cerebellar Network Shifting in Spinocerebellar Ataxia Type 6. Cereb Cortex. 2016;26(7):3205–18. 10.1093/cercor/bhv154. PMC4898673 26209844

[44] Pedroso JL, de Carvalho Campos-Neto G, Speciali DS, Barsottini OG, Bor-Seng-Shu E, Felicio AC. Spinocerebellar ataxia type 6 presenting with parkinsonism, pre-synaptic dopaminergic dysfunction and hyperechogenicity of the substantia nigra. J Neurol Sci. 2017;376:60–2. 10.1016/j.jns.2017.02.038. 28431629

[45] Rueda AD, Pedroso JL, Truksinas E, Do Prado GF, Coelho FM, Barsottini OG. Polysomnography findings in spinocerebellar ataxia type 6. J Sleep Res. 2016;25(6):720–3. 10.1111/jsr.12439. 27377205

[46] Rezende TJR, Adanyaguh I, Barsottini OGP, Bender B, Cendes F, Coutinho L, et al. Genotype-specific spinal cord damage in spinocerebellar ataxias: an ENIGMA-Ataxia study. J Neurol Neurosurg Psychiatry. 2024;95(7):682–90. 10.1136/jnnp-2023-332696. PMC11187354 38383154

[47] de Borba FC, Fernandes JMS, de Rezende TJR, González-Salazar C, de Melo Teixeira Branco L, Wolmer PS, et al. Tract-specific spinal damage in SCA2, SCA3 and SCA6. J Neurol. 2024;272(1):6. 10.1007/s00415-024-12791-6. 39666101

[48] O’Donnell LJ, Westin CF. An introduction to diffusion tensor image analysis. Neurosurg Clin N Am. 2011;22(2):185–96, viii. 10.1016/j.nec.2010.12.004 PMC3163395 21435570

[49] Winklewski PJ, Sabisz A, Naumczyk P, Jodzio K, Szurowska E, Szarmach A. Understanding the Physiopathology Behind Axial and Radial Diffusivity Changes-What Do We Know? Front Neurol. 2018;9:92. 10.3389/fneur.2018.00092. PMC5835085 29535676

[50] Jacobi H, Bauer P, Giunti P, Labrum R, Sweeney MG, Charles P, et al. The natural history of spinocerebellar ataxia type 1, 2, 3, and 6: a 2-year follow-up study. Neurology. 2011;77(11):1035–41. 10.1212/WNL.0b013e31822e7ca0. PMC3174068 21832228

[51] Figley CR, Uddin MN, Wong K, Kornelsen J, Puig J, Figley TD. Potential Pitfalls of Using Fractional Anisotropy, Axial Diffusivity, and Radial Diffusivity as Biomarkers of Cerebral White Matter Microstructure. Front Neurosci. 2021;15:799576. 10.3389/fnins.2021.799576. PMC8795606 35095400

[52] Genc S, Malpas CB, Holland SK, Beare R, Silk TJ. Neurite density index is sensitive to age related differences in the developing brain. NeuroImage. 2017;148:373–80. 10.1016/j.neuroimage.2017.01.023. 28087489

[53] Guerrini L, Lolli F, Ginestroni A, Belli G, Della Nave R, Tessa C, et al. Brainstem neurodegeneration correlates with clinical dysfunction in SCA1 but not in SCA2. A quantitative volumetric, diffusion and proton spectroscopy MR study. Brain. 2004;127(Pt 8):1785–95. 10.1093/brain/awh201. 15240431

[54] Manto M, Bower JM, Conforto AB, Delgado-García JM, da Guarda SNF, Gerwig M, et al. Consensus paper: roles of the cerebellum in motor control–the diversity of ideas on cerebellar involvement in movement. Cerebellum. 2012;11(2):457–87. 10.1007/s12311-011-0331-9. PMC4347949 22161499

[55] Dum RP, Strick PL. An unfolded map of the cerebellar dentate nucleus and its projections to the cerebral cortex. J Neurophysiol. 2003;89(1):634–9. 10.1152/jn.00626.2002. 12522208

[56] Klinke I, Minnerop M, Schmitz-Hübsch T, Hendriks M, Klockgether T, Wüllner U, et al. Neuropsychological features of patients with spinocerebellar ataxia (SCA) types 1, 2, 3, and 6. Cerebellum. 2010;9(3):433–42. 10.1007/s12311-010-0183-8. PMC2949561 20502998

[57] McGaugh JL. Memory consolidation and the amygdala: a systems perspective. Trends Neurosci. 2002;25(9):456. 10.1017/S1355617716000084. 12183206

[58] Hernandez-Castillo CR, Vaca-Palomares I, Galvez V, Campos-Romo A, Diaz R, Fernandez-Ruiz J. Cognitive Deficits Correlate with White Matter Deterioration in Spinocerebellar Ataxia Type 2. J Int Neuropsychol Soc. 2016;22(4):486–91. 10.1017/S1355617716000084. 26888086

